# Efficacy and Safety of Treating Refractory Bone and Soft Tissue Sarcoma with Anlotinib in Different Treatment Patterns

**DOI:** 10.1155/2022/3287961

**Published:** 2022-08-11

**Authors:** Meng Cai, Jie Zhu, Guangxin Zhou

**Affiliations:** ^1^Department of Orthopedics, Jinling Hospital, Nanjing University School of Medicine, Nanjing, Jiangsu, China; ^2^School of Medicine, Southeast University, Nanjing, Jiangsu, China; ^3^Department of Painology, Jinling Hospital, Nanjing University School of Medicine, Nanjing, Jiangsu, China

## Abstract

**Methods:**

The medical data of 47 patients with refractory bone and soft tissue sarcoma, who received anlotinib from January 2019 to December 2020, were retrospectively collected. The overall response rate (ORR) and disease control rate (DCR) were evaluated according to the solid tumor response evaluation version 1.1 standard. The progression-free survival (PFS), overall survival (OS), and adverse reactions were recorded.

**Results:**

A total of 44 patients, including 13 with osteosarcoma and 31 with soft tissue sarcoma, were enrolled in this study. Among patients with osteosarcoma, no patients achieved complete response (CR) or partial response (PR), while seven patients (54%) had stable disease (SD). Besides, the median PFS (m-PFS) was 4.4 months, and the median OS (m-OS) was 15.7 months. Among patients with soft tissue sarcoma, the ORR and DCR were 19% and 71%, respectively. The median m-PFS was 5.4 months, and m-OS was 17.9 months. Anlotinib plus chemotherapy had a higher ORR compared with anlotinib monotherapy (6% vs. 38%, *P* = 0.047). The most common grade 3/4 adverse reactions were pneumothorax (5%) and pleural effusion (5%), and no treatment-related deaths occurred.

**Conclusions:**

Anlotinib alone showed encouraging efficacy and favorable tolerability in refractory bone and soft tissue sarcoma. Anlotinib plus chemotherapy did not show a significant clinical benefit compared with anlotinib alone. Anlotinib showed better tumor control when used as first-line drug treatment in refractory bone and soft tissue sarcoma.

## 1. Introduction

Bone and soft tissue sarcomas (STSs) are rare mesenchymal malignancies with a high histological heterogeneity [1]. Up to 50% of patients develop metastasis, which may appear either as *de novo* or recurrent advanced disease [2]. Patients with recurrence or metastasis are often resistant to chemotherapy [3, 4]. Up to 50% of patients will develop metastasis, which may appear as either de novo or recurrent advanced disease [2]. Patients with recurrence or metastasis are often resistant to chemotherapy [4]. Therefore, it is critical to explore more effective therapeutic strategies for patients with metastatic or recurrent bone and soft tissue sarcoma.

Anlotinib is a novel multitarget tyrosine kinase inhibitor against both tumor

angiogenesis and tumor cell proliferation through blocking vascular endothelial growth factor receptor, fibroblast growth factor receptor, and stem cell factor receptor c-Kit [5]. Based on the unique cyclopropyl structure, the half-maximal inhibitory concentration (IC50) of anlotinib for each target is low, which contributes to the inhibition of angiogenesis and tumor growth and development of less drug resistance and side effects compared with other single-target drugs [6]. In China, anlotinib obtained the initial approval by the China State Food and Drug Administration for patients with advanced-stage non-small-cell lung cancer (NSCLC) after receiving two lines of chemotherapy in 2018 [7]. Numerous clinical studies showed that anlotinib displayed promising efficacy and safety in various cancer types such as small-cell lung cancer, cervical cancer, high-grade glioma, and desmoid tumor [6, 8–10]. Moreover, anlotinib showed encouraging antitumor activity in patients with unresectable or metastatic bone sarcoma [11].

Due to the pharmacokinetics of the drug, anlotinib can be combined with cytotoxic drugs with limited drug–drug interactions. Compared with chemotherapy alone, anlotinib plus chemotherapy has shown promising efficacy in terms of progression-free survival (PFS) and overall survival (OS) in multiple tumor types such as advanced-stage non-small-cell lung cancer, ovarian cancer, and renal cancer [12]. For example, a phase I study suggested that the combination of anlotinib with platinum/pemetrexed-based chemotherapy had good efficacy and tolerance among patients with advanced nonsquamous NSCLC, which was used as a first-line regimen [13]. Moreover, further research indicated that anlotinib plus chemotherapy was a promising strategy in terms of the DCR, median OS, and tolerance for patients with advanced-stage NSCLC after failing first- or second-line therapy [14]. However, the clinical efficacy of anlotinib plus chemotherapy for bone and STS is still uncertain.

Therefore, we conducted a retrospective single-center analysis to compare the similarities and differences in patients with refractory sarcoma who received different treatment options.

## 2. Materials and Methods

### 2.1. Patient Population and Data Collection

We retrospectively analyzed the electronic medical records of 47 patients who received anlotinib from January 2019 to December 2020 at Jinling Hospital (Nanjing, China). The eligibility criteria were as follows: (1) histologically confirmed osteosarcoma or STS, (2) tumor recurrence or metastasis after treatment, (3) received at least one cycle of anlotinib therapy without other targeted drugs, and (4) adequate hepatic, cardiac, hematologic, and renal function. Patients were excluded when they received other targeted drugs. This study was approved by the ethics committee. The requirement for obtaining informed consent was waived given the study was retrospective.

The pathological subtypes of STS included in our study were fibrosarcoma (FS), synovial sarcoma (SS), pleomorphic undifferentiated sarcoma (UPS), leiomyosarcoma (LMS), liposarcoma (LPS), clear cell sarcoma, alveolar soft part sarcoma (ASPS), rhabdomyosarcoma, angiosarcoma (AS), epithelioid sarcoma (ES), and so forth.

### 2.2. Treatment

All patients received anlotinib at an initial dose of 12 mg once daily from days 1 to 14 every 3 weeks until disease progression or intolerable toxicity. The initial dose of anlotinib was allowed to be reduced to 10 mg or 8 mg when unacceptable adverse events (AEs) occurred. AEs were classified and evaluated according to the National Cancer Institute Common Terminology Criteria for Adverse Events, version 4.0.

### 2.3. Efficacy Evaluation

The tumor response was evaluated using the solid tumor efficacy evaluation

standard (RECIST 1.1), which included complete response (CR), partial response (PR), stable disease (SD), and progressive disease (PD). In addition, overall response rate (ORR), disease-control rate (DCR), the median progression-free survival (PFS), and overall survival (OS), during anlotinib maintenance, were recorded and analyzed. The efficacy in patients treated with apatinib was calculated as previously described [6].

### 2.4. Statistical Analysis

All statistical analyses were conducted using SPSS version 21 (IBM, NC, USA) or GraphPad Prism 8.0 (GraphPad, CA, USA). The clinical characteristics of patients were described in terms of the median (95% confidence interval) or frequency (percentage). The survival of patients (m-PFS and OS) was estimated using the Kaplan–Meier method and the log-rank test. Subsequently, the survival curves were generated according to Prism 8.0. A value of *P* < 0.05 indicated a statistically significant difference.

## 3. Results

### 3.1. Patient Characteristics

Between January 2019 and December 2020, 47 patients who underwent anlotinib treatment at Jinling Hospital (Nanjing, China) were initially included in the study. The last interview ended in June 2021, and the average follow-up time was 13.6 months. Finally, 3 patients were lost to follow-up, and 44 patients, including 13 with osteosarcoma and 31 with STS, were included. The baseline characteristics of patients with refractory bone and STS are summarized in Table 1. The median age was 48.5 years (range: 8–76) years, and 21 patients were male. The histological subtypes of STS are presented in Supplementary Table 1. Further, 30 patients had a good performance status (Eastern Cooperative Oncology Group (ECOG) 0–1). Of the 44 patients, 82% (36/44) received primary surgery, 57% (25/44) received chemotherapy, and 18% (8/44) received radiotherapy. Local recurrence occurred in 25 patients, and distant metastasis (mainly of the lung) was discovered in 23 patients. Also, 55% (24/44) patients underwent treatment with anlotinib alone, 41% (18/44) received anlotinib plus chemotherapy, and 5% (2/44) received anlotinib plus radiotherapy. The chemotherapy regimen applied for osteosarcoma patients mainly based on high-dose methotrexate, cisplatin, doxorubicin, and ifosfamide. The chemotherapy regimen applied for STS patients included anthracyclines, ifosfamide, dacarbazine, docetaxel, temozolomide, epirubicin, gemcitabine, cyclophosphamide, and vincristine. Two patients received stereotactic body radiotherapy and dose ranged from 15 to 60 Gy in 1-8 fractions in one treatment course. Besides, anlotinib was used as first-line therapy in 10 patients, second-line therapy in 26 patients, and beyond second-line therapy in 26 patients.

## 4. Efficacy

**Figure 1:**
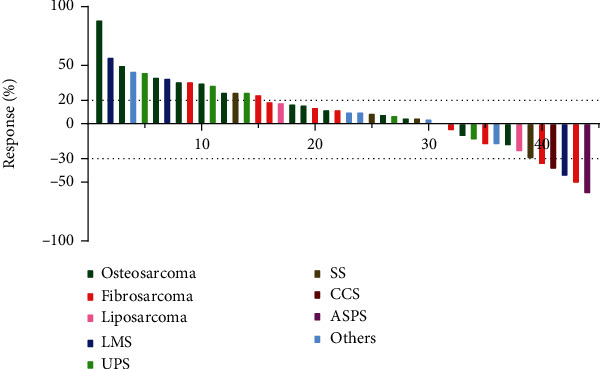
Waterfall plots for maximum changes in sizes of target lesions versus baseline during anlotinib treatment.

The tumor response conditions are presented in Figure 1. Although none achieved CR, 6 patients had PR, 23 patients had SD, and 15 patients had PD, with an ORR of 14% and a DCR of 66%.

### 4.1. Osteosarcoma

**Figure 2:**
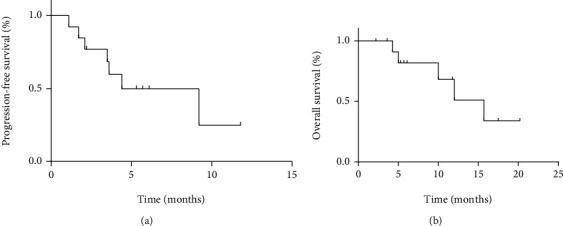
(a, b) Kaplan–Meier curve of progression-free survival and overall survival for patients with osteosarcoma.

For all patients with osteosarcoma, no CR or PR but seven SD and six PD were recorded, with a DCR of 54% (Table 2). The m-PFS and median OS (m-OS) were 4.4 months (95% CI: 0–9.1 months) and 15.7 months (95% CI: 9.5–21.9 months), respectively (Figure 2).

**Figure 3:**
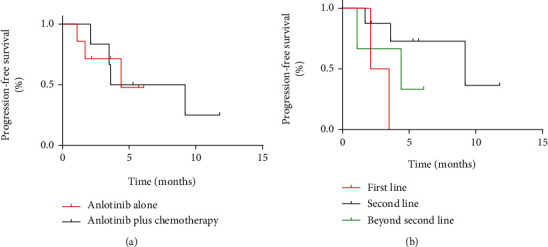
(a, b) Kaplan–Meier curve of progression-free survival.

No significant difference was found in the DCR (57% vs. 50%, *P* = 0.617) and m-PFS (4.4 months vs. 3.6 months, *P* = 0.959) between anlotinib alone and anlotinib plus chemotherapy groups. Patients who received anlotinib as first-line therapy combined with MAP standard chemotherapy achieved PD. A total of three patients received anlotinib alone as beyond second-line therapy. Two patients achieved SD, and one patient achieved PD (Table 2). No statistically significant difference was observed in DCR between different treatment lines, while the m-PFS of second-line therapy was significantly higher than that of first-line therapy (*P* = 0.033) (Figure 3).

### 4.2. Soft Tissue Sarcoma

**Figure 4:**
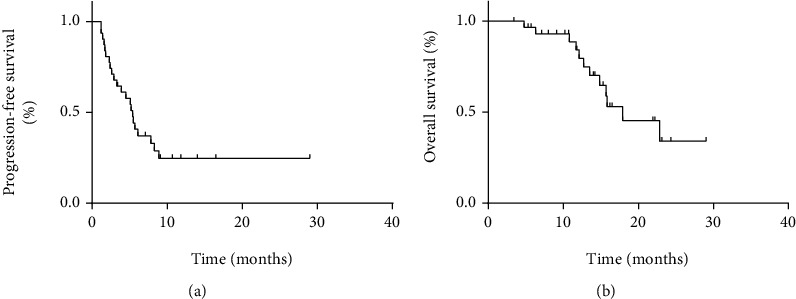
(a, b) Kaplan–Meier curve of progression-free survival and overall survival for patients with soft tissue sarcoma.

**Figure 5:**
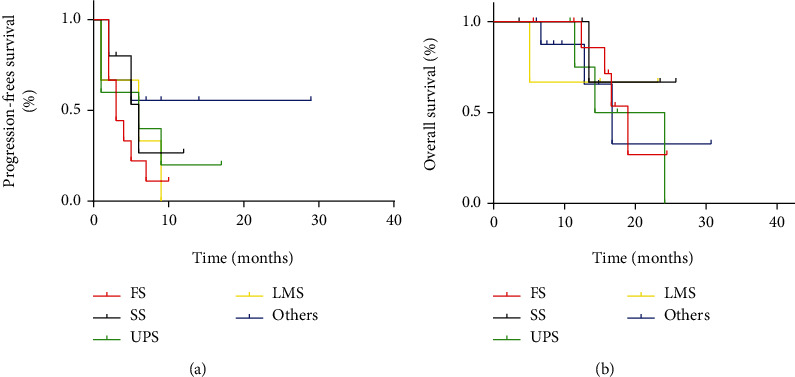
Kaplan–Meier curve of progression-free survival and overall survival for patients with soft tissue sarcoma of different subtypes.

Although none achieved CR, 6 PR, 16 SD, and 9 PD were observed in patients with STS. The ORR and DCR were 19% and 52%, respectively. Besides, m-PFS and m-OS were 5.4 months (95% CI: 4.1–6.8 months) and 17.9 months (95% CI: 11.2–24.6 months), respectively (Figure 4). The clinical benefits and outcomes of patients with different soft tissue sarcoma subtypes were different, which are presented in Tables 3 and 4 and Figure 5.

**Figure 6:**
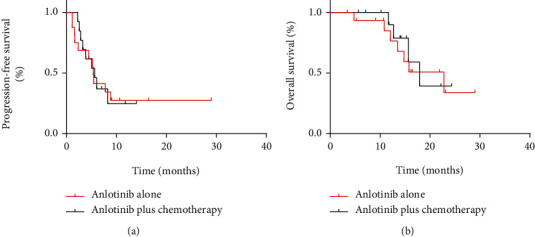
(a, b) Kaplan–Meier curve of progression-free survival and overall survival for patients with soft tissue sarcoma receiving anlotinib alone and anlotinib plus chemotherapy or radiotherapy.

A total of 4 patients achieved PR, 11 patients achieved SD, and 4 patients achieved PD in the anlotinib-alone group; 5 with PR, 4 with SD, and 4 with PD were found in the anlotinib plus chemotherapy groups (Table 5). For patients in the two groups, the ORR and DCR were 6% versus 38% (*P* = 0.047) and 75% versus 69% (*P* = 0.526), respectively. Besides, m-PFS and m-OS were 5.4 months versus 5.7 months (*P* = 0.936) and 22.8 months versus 17.9 months, respectively (*P* = 0.747) (Table 6 and Figure 6). For two patients receiving anlotinib plus radiotherapy, one with SD and one with PD were found.

**Figure 7:**
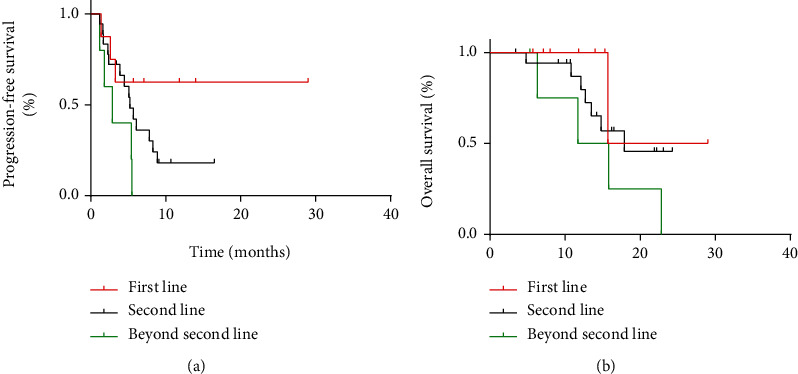
(a, b) Kaplan–Meier curve of progression-free survival and overall survival for patients with soft tissue sarcoma who used anlotinib as first-line therapy, second-line therapy, or beyond second-line therapy.

Among patients who received anlotinib as first-line therapy, four patients achieved PR and four patients achieved SD. Among patients who received anlotinib as second-line therapy, two patients achieved PR, nine patients achieved SD, and seven patients achieved PD. Among patients who received anlotinib as beyond second-line therapy, three patients achieved SD and two patients achieved PD. The result indicated that the ORR and DCR were 50% and 100%, respectively, which were significantly higher than others (ORR 50% vs. 9%, *P* = 0.026; DCR 100% vs. 61%, *P* = 0.041). However, the m-PFS and m-OS of the different lines were similar (Figure 7).

## 5. Safety

As shown in Table 7, 35 (79.5%) patients experienced grade 1 or 2 AEs, including hand-foot syndrome (12, 27%), diarrhea (8, 18%), hoarseness or sore throat (7, 16%), vomiting (4, 9%), hypertension (3, 7%), and pneumothorax (1, 2%). Grade 3/4 AEs were hand-foot syndrome (1, 2%), hypertension (1, 2%), anemia (1, 2%), hemoptysis (1, 2%), pleural effusion (2, 5%), and pneumothorax (2, 5%). The incidence of adverse reactions in monotherapy patients was 52% (12/23), with two cases (9%) of grade 3/4 adverse reactions. The incidence of adverse reactions in patients with combined chemotherapy was 53% (10/19), with three cases (16%) of grade 3/4 adverse reactions. A permanent dose reduction was conducted in 3 patients: to 10 mg/day in 2 (45.5%) patients and to 8 mg/day in 1 (22.7%) patient. Most of them underwent three (range, 1-14) treatment cycles before treatment was discontinued because of the intolerable toxicity.

## 6. Discussion

Over the past few decades, little progress has been made to treat bone and STSs; surgery and chemotherapy are still the most important options [15]. For patients with refractory sarcoma, the efficacy of conventional standard treatment is not satisfactory, and more effective treatment methods are urgently needed. Our study retrospectively analyzed the efficacy and safety of different treatment modalities for anlotinib in refractory bone and STSs.

The current therapeutic strategies for patients with recurrent or metastatic osteosarcoma, who failed first-line standard therapy, have limited efficacy, and the 5-year survival rate of patients is less than 30% [16]. Some small-molecule antiangiogenic tyrosine kinase inhibitors (TKI), such as sorafenib, regorafenib, and pazopanib, have been used for treating refractory osteosarcoma and shown encouraging antitumor activity (m-PFS, 3.6–6 months) [17–20]. The m-PFS of patients with refractory osteosarcoma in our study was 4.4 months, which was consistent with that reported in the studies mentioned earlier. Although seven patients achieved SD and six patients achieved PD, no CR or PR was observed. Even extensive antitumor activity induced by anlotinib might not result in remarkable tumor shrinkage due to the abundant bone matrix contained in osteosarcoma [21].

Wang et al. in their study observed that anlotinib exhibited potent inhibitory effects in the orthotopic osteosarcoma xenograft model and preclinical osteosarcoma patient-derived xenograft model significantly repressed tumor growth, metastasis, and angiogenesis. Besides, they found that anlotinib could enhance the chemosensitivity of osteosarcoma cells to cisplatin (DDP), and the combination of anlotinib with DDP significantly reduced tumor size compared with either anlotinib or DDP alone [22]. Subsequently, further research demonstrated that anlotinib might be used to reverse the multidrug resistance of doxorubicin (DOX) [23]. Both DDP and DOX are included in the standard chemotherapy regimen for osteosarcoma. Therefore, anlotinib plus chemotherapy may be an effective treatment option for patients with osteosarcoma. In our study, no significant differences were observed in tumor response and survival prognosis between the combination chemotherapy and single-agent groups. The two patients who received anlotinib plus chemotherapy as first-line therapy were diagnosed with high-grade osteosarcoma and had large tumor size (>5 cm), which led to preferring amputation rather than limb salvage. Moreover, the treatment for osteosarcoma was inevitably subject to selection bias. This might contribute to a lower m-PFS in patients receiving anlotinib as first-line therapy compared with second-line therapy and narrow the difference in efficacy between the single-agent and combination chemotherapy groups.

Until now, several clinical trials have been already in progress, and new trials will begin within the next few years. For example, a single-arm multicenter trial of anlotinib plus chemotherapy in patients with Stage IIB classic osteosarcoma of the extremity is expected to be completed by 2025 (ChiCTR2000033298). Preliminary results from another study revealed the clinical efficacy of anlotinib combined with irinotecan and vincristine in advanced-stage Ewing sarcoma, with an ORR of 62.5% in adults and 83.3% in children after 12 weeks (NCT03416517). Additionally, a study of anlotinib combined with or without PD-1 antibody for unresectable high-grade chondrosarcoma is enrolling patients (NCT05193188).

STS is relatively rare and accounts for less than 1% of adult cancers with an estimated 12,000 new cases in the USA each year [24]. The current standard treatment for STS consists of extensive surgical excision with or without chemotherapy [25]. The prognosis of refractory soft tissue sarcomas remains poor, with a median OS of 8–12 months [26]. The clinical efficacy and safety of anlotinib in soft tissue sarcoma have been demonstrated in previous clinical trials [5, 27]. In our study, the ORR and DCR were 19% and 71%, respectively. Besides, m-PFS and m-OS were 5.4 months and 17.9 months, respectively.

Different TKI drugs have shown particular effects in different subtypes of soft tissue sarcoma [28]. Pazopanib showed significant antitumor activity in leiomyosarcomas and synovial sarcomas [29, 30], while sorafenib exhibited good tolerability and promising antitumor activity in treating angiosarcoma [31]. The study by Chi et al. discovered that several subtypes of soft tissue sarcoma, including fibrosarcoma, synovial sarcoma, liposarcoma, and acinar soft tissue sarcoma, exhibited a higher sensitivity to anlotinib compared with other multikinase inhibitors [27]. In the present study, we observed that patients with fibrosarcoma, synovial sarcoma, and liposarcoma had significantly higher ORR or DCR than patients with other pathological subtypes. Anlotinib showed a significant effect in a patient with alveolar soft part sarcoma and a patient with clear cell sarcoma. Both of them achieved CR and had longer PFS (29 months and 20 months, respectively). Clear cell sarcoma is a rare malignant soft tissue tumor with few effective treatments [32]. Our study revealed that anlotinib might be an effective treatment option for clear cell sarcoma.

A few clinical trials have been conducted on TKIs and chemotherapy combination therapy, and even fewer are related to anlotinib. A small retrospective study involving 32 patients with advanced/metastatic STS showed that anlotinib plus chemotherapy remarkably improved survival prognosis (ORR 34%, m-PFS 8.2 months) compared with chemotherapy alone [33]. Moreover, another study indicated that anlotinib combined with liposomal doxorubicin following anlotinib maintenance could be used as an effective treatment for patients with metastatic STS, with an ORR of 40.7% and an m-PFS time of 7 months [34]. In this study, five patients achieved PR, four patients achieved SD, and four patients achieved PD, with an ORR of 38% and an m-PFS time of 5.7 months. We observed that anlotinib plus chemotherapy compared with anlotinib significantly improved the ORR (6% vs. 38%, *P* = 0.047), but with no statistically significant difference in DCR, m-PFS, and m-OS between the two groups. Similarly, a multicenter study involving 76 patients with metastatic STS reported that anlotinib combined with doxorubicin improved the ORR but not PFS in patients with STS [35]. This result might be associated with the drug resistance to anlotinib. Thus, anlotinib plus neoadjuvant chemotherapy could be used for patients with difficult surgery to narrow the tumor volume, reduce functional damage, and improve quality of life. Besides, the ORR and DCR of first-line therapy were significantly higher than those of other later-line treatments. Among eight patients who received anlotinib as first-line treatment, six patients received the combination of anlotinib plus chemotherapy, which may had some impact on the results.

Anlotinib compared with other TKIs was well tolerated, and most adverse reactions were not serious and reversible. The incidence of adverse reactions in patients with combination chemotherapy was similar to that in patients who underwent anlotinib alone. Pneumothorax is a common adverse response for patients with sarcoma treated with TKIs. A retrospective study of 47 patients with relapsed STS receiving pazopanib reported that pneumothorax occurred in 9 patients (9%), and 8 patients reached grade 3/4 [36]. Besides, a previous study indicated that the incidence of pneumothorax in treating advanced osteosarcoma with anlotinib was 32.4% (12 cases), and six of them reached grade 3/4 [20]. In our study, we found that severe pneumothorax occurred in three patients with pulmonary metastases. Subsequently, two patients with grade 3/4 pneumothorax received closed thoracic drainage. The occurrence of pneumothorax might be related to lung metastases from sarcoma [37]. Progression or necrosis of lung metastases can cause pulmonary reactions such as pneumothorax.

However, this study had some shortcomings. Our study was retrospective and the sample size was small, leading to selection bias. Besides, the follow-up time was not long enough to explore the clinical outcomes of patients receiving different treatments.

## 7. Conclusions

Collectively, our results indicated that anlotinib had broad-spectrum antitumor activity against refractory bone and soft tissue sarcomas. For patients with osteosarcoma, anlotinib plus chemotherapy did not show a significant therapeutic efficacy compared with that of anlotinib alone in treating osteosarcoma. Besides, anlotinib might be a good option as a second-line drug after the failure of first-line therapy. For patients with soft tissue sarcomas, anlotinib plus chemotherapy did not bring significant clinical benefit. However, the combination regimen brings higher ORR compared with anlotinib alone. Besides, anlotinib showed better tumor control (higher ORR and DCR) when used as first-line drug treatment. Thus, anlotinib plus chemotherapy used as first-line therapy may be an effective treatment for refractory bone and soft tissue sarcoma. Most toxicities were manageable. It is vital to further explore the clinical benefit of anlotinib in more prospective trials.

## Figures and Tables

**Table 1:** Patient characteristics.

Characteristics	Osteosarcoma (*n* = 13)	Soft tissue sarcoma (*n* = 31)
Age, median (range)	28 (8–62)	55 (19–76)
Sex, *n* (%)		
Male	9 (69)	12 (39)
Female	4 (31)	19 (61)
ECOG performance status, *n* (%)		
0–1	10 (77)	20 (64)
2–3	3 (23)	11 (36)
Primary tumor site, *n* (%)		
Extremities	12 (92)	21 (68)
Trunk	1 (8)	10 (32)
Surgical history, *n* (%)		
Yes	11 (85)	25 (81)
No	2 (15)	6 (19)
Chemotherapy history, *n* (%)		
Yes	13 (100)	12 (39)
No	0 (0)	19 (61)
Radiotherapy history, *n* (%)		
Yes	0 (0)	8 (26)
No	13 (100)	23 (74)
Recurrence, *n* (%)		
Yes	8 (62)	21 (68)
No	5 (38)	10 (32)
Distant metastases, *n* (%)		
Lung only	8 (62)	10 (32)
Other single	0 (0)	3 (10)
Multiple organs	2 (15)	2 (6)
Therapy, *n* (%)		
Anlotinib alone	7 (54)	16 (52)
Anlotinib plus chemotherapy	6 (46)	13 (42)
Anlotinib plus radiotherapy	0 (0)	2 (6)
Treatment lines of anlotinib, *n* (%)		
First line	2 (15)	8 (26)
Second line	8 (62)	18 (58)
Beyond second line	3 (23)	5 (16)

ECOG: Eastern Cooperative Oncology Group.

**Table 2:** DCR of patients with osteosarcoma using anlotinib as first-line therapy, second-line therapy, or beyond second-line therapy.

Treatment line	DCR		
	Anlotinib	Anlotinib plus chemotherapy	Total
First line	0 (0/0)	0 (0/2)	0 (0/2)
Second line	50 (2/4)	75 (3/4)	63 (5/8)
Beyond second line	67 (2/3)	0 (0/0)	67 (2/3)
Total	57 (4/7)	50 (3/6)	54 (7/13)

**Table 3:** Responses of various histological subtypes to anlotinib.

Histological subtype	CR *n* (%)	PR *n* (%)	SD *n* (%)	PD *n* (%)	ORR *n* (%)	DCR *n* (%)
FS	0 (0)	2 (22)	5 (56)	2 (22)	2 (22)	7 (78)
SS	0 (0)	1 (20)	3 (60)	1 (20)	1 (20)	4 (80)
UPS	0 (0)	0 (0)	2 (40)	3 (60)	0 (0)	2 (40)
LMS	0 (0)	1 (33)	0 (0)	2 (67)	1 (33)	1 (33)
LPS	0 (0)	0 (0)	2 (100)	0 (0)	0 (0)	2 (100)
CCS	0 (0)	1 (100)	0 (0)	0 (0)	1 (100)	1 (100)
ASPS	0 (0)	1 (100)	0 (0)	0 (0)	1 (100)	1 (100)
Others	0 (0)	0 (0)	4 (80)	1 (20)	0 (0)	4 (80)
Total	0 (0)	6 (19)	16 (52)	9 (29)	6 (19)	22 (71)

**Table 4:** Median survival of patients with soft tissue sarcoma having different histologic subtypes.

	Progression-free survival (month)		Overall survival (month)	
	Median	95% CI	Median	95% CI
FS	3.9	2.1–5.7	17.9	15.0–20.8
SS	5.7	2.9–8.5	—	—
UPS	5.4	0–13.5	13.5	5.7–21.3
LMS	6.1	0–13.3	—	—
Others	—	—	15.8	10.2–21.4
Total	5.4	4.1–6.8	17.9	11.2–24.6

**Table 5:** OCR/DCR of patients with soft tissue sarcoma using different treatment methods.

Treatment	Anlotinib	Anlotinib plus chemotherapy	Anlotinib plus radiotherapy	Total
First line (OCR and DRR)	100 (1/1), 100 (1/1)	50 (3/6), 100 (6/6)	0 (0/1), 100 (1/1)	50 (4/8), 100 (8/8)
Second line (OCR and DRR)	0 (0/12), 67 (8/12)	33 (2/6), 50 (3/6)	0 (0/0), 0 (0/0)	11 (2/18), 61 (11/18)
Beyond second line (OCR and DRR)	0 (0/3), 100 (3/3)	0 (0/1), 0 (0/1)	0 (0/1), 0 (0/1)	0 (0/5), 60 (3/5)
Total	6 (1/16), 75 (12/16)	38 (5/13), 69 (9/13)	0 (0/2), 50 (1/2)	19 (6/31), 71 (22/31)

**Table 6:** Median survival of patients with soft tissue sarcoma receiving different treatments.

Treatment	Progression-free survival (month)		Overall survival (month)	
	Median	95% CI	Median	95% CI
First line	—	—	15.7	—
Second line	5.2	3.6–6.8	17.9	—
Beyond second line	2.9	0.5–5.3	11.7	2.4–21.0
Anlotinib	5.4	4.9–6.0	22.8	12.4–33.2
Anlotinib plus chemotherapy	5.7	3.3–8.1	17.9	13.3–22.5
Anlotinib plus radiotherapy	1.4	—	6.3	—
Total	5.4	4.1–6.8	17.9	11.2–24.6

**Table 7:** Treatment-related adverse events (*n*, %).

AEs	Grades 1–2	Grades 3–4	Total
Hand-foot syndrome	12 (27)	1 (2)	13 (30)
Diarrhea	8 (18)	0 (0)	8 (18)
Hoarseness or sore throat	7 (16)	0 (0)	7 (16)
Vomit	4 (9)	0 (0)	4 (9)
Hypertension	3 (7)	1 (2)	4 (9)
Pleural effusion	0 (0)	2 (5)	2 (5)
Pneumothorax	1 (2)	2 (5)	3 (7)
Anemia	0 (0)	1 (2)	1 (2)
Hemoptysis	0 (0)	1 (2)	1 (2)

## Data Availability

The date included in this study are present in the article. The origin data are available upon reasonable requests from the corresponding author.

## Supplementary Materials

Supplementary Materials Supplementary Table 1: histological subtype of STS is presented.
