# Supplementary material for: Efficacy and Safety of Treating Refractory Bone and Soft Tissue Sarcoma with Anlotinib in Different Treatment Patterns
Source: Comput Math Methods Med. 2022 Aug 11;2022:3287961. doi: 10.1155/2022/3287961 (PMC9388280; doi:10.1155/2022/3287961)
Supplement: Supplementary Materials — Supplementary Table 1: histological subtype of STS is presented. [file 3287961.f1.docx]

**Supplementary Table 1. Histological subtype of STS**

| **Histological subtype** | ***n* (%)** |
| --- | --- |
| **FS** | 9 (29) |
| **SS** | 5 (16) |
| **UPS** | 5 (16) |
| **LMS** | 3 (10) |
| **LPS** | 2 (6) |
| **CCS** | 1 (3) |
| **ASPS** | 1 (3) |
| **AS** | 1 (3) |
| **ES** | 1 (3) |
| **Primitive neuroectodermal tumor** | 1 (3) |
| **Rhabdomyosarcoma** | 1 (3) |
| **Unspecified sarcoma** | 1 (3) |
